# The Major Birch Pollen Allergen Bet v 1 Induces Different Responses in Dendritic Cells of Birch Pollen Allergic and Healthy Individuals

**DOI:** 10.1371/journal.pone.0117904

**Published:** 2015-01-30

**Authors:** Ursula Smole, Christian Radauer, Nina Lengger, Martin Svoboda, Neil Rigby, Merima Bublin, Sonja Gaier, Karin Hoffmann-Sommergruber, Erika Jensen-Jarolim, Diana Mechtcheriakova, Heimo Breiteneder

**Affiliations:** 1 Department of Pathophysiology and Allergy Research; Center for Pathophysiology, Infectiology, and Immunology, Medical University of Vienna, Vienna, Austria; 2 Department of Environmental Health Sciences, Bloomberg School of Public Health, Johns Hopkins University, Baltimore, Maryland, United States of America; 3 Institute of Food Research, Norwich Research Park, Norwich, United Kingdom; 4 Messerli Research Institute of the Medical University of Vienna, Veterinary University of Vienna and University of Vienna, Austria; Tulane University, UNITED STATES

## Abstract

Dendritic cells play a fundamental role in shaping the immune response to allergens. The events that lead to allergic sensitization or tolerance induction during the interaction of the major birch pollen allergen Bet v 1 and dendritic cells are not very well studied. Here, we analyzed the uptake of Bet v 1 and the cross-reactive celery allergen Api g 1 by immature monocyte-derived dendritic cells (iMoDCs) of allergic and normal donors. In addition, we characterized the allergen-triggered intracellular signaling and transcriptional events. Uptake kinetics, competitive binding, and internalization pathways of labeled allergens by iMoDCs were visualized by live-cell imaging. Surface-bound IgE was detected by immunofluorescence microscopy and flow cytometry. Allergen- and IgE-induced gene expression of early growth response genes and Th1 and Th2 related cytokines and chemokines were analyzed by real-time PCR. Phosporylation of signaling kinases was analyzed by Western blot. Internalization of Bet v 1 by iMoDCs of both donor groups, likely by receptor-mediated caveolar endocytosis, followed similar kinetics. Bet v 1 outcompeted Api g 1 in cell surface binding and uptake. MoDCs of allergic and healthy donors displayed surface-bound IgE and showed a pronounced upregulation of Th2 cytokine- and NFκB-dependent genes upon non-specific Fcε receptor cross-linking. In contrast to these IgE-mediated responses, Bet v 1-stimulation increased transcript levels of the Th2 cytokines IL-4 and IL-13 but not of NFκB-related genes in MoDCs of BP allergic donors. Cells of healthy donors were either unresponsive or showed elevated mRNA levels of Th1-promoting chemokines. Moreover, Bet v 1 was able to induce Erk1/2 and p38 MAPK activation in BP allergics but only a slight p38 activation in normal donors. In conclusion, our data indicate that Bet v 1 favors the activation of a Th2 program only in DCs of BP allergic individuals.

## Introduction

Type 1 allergic diseases are mediated by antibodies of the IgE class that are induced by otherwise innocuous environmental substances in genetically predisposed individuals. Type 1 allergies affect 300–400 million people worldwide, generate 120 billion Euros in health care costs, sick leave and economic losses, and significantly reduce the quality of life for the allergic individual [1]. Although detailed information is available on the various protein allergens, very little is known about how these molecules initiate allergic sensitization.

In recent years, the interest in innate immune cells as important regulators of adaptive immune responses to allergens has increased considerably [2]. For example, pattern recognition receptors, which are expressed by all innate immune cells and detect conserved molecular patterns on invading pathogens, are also able to recognize allergen-associated molecular signatures [3, 4]. Consequently, considerable effort has been made to identify distinct molecular features of allergens that trigger innate immune pathways. Protein conformation [5–7], lipid-binding ability and activation of toll-like receptors [4, 8], presence of carbohydrate structures that bind to C-type lectins [3, 9], and protease activity [10, 11] may contribute to the allergenic potential of a protein. In addition to allergen-specific characteristics, external factors such as environmental pollutants [12, 13] and matrix components of the allergen source [4, 14, 15] can promote a Th2-favoring milieu via their immunomodulatory and adjuvant activities.

Notably, the majority of the population does not develop allergies despite being exposed to allergenic proteins. This may point to allergen-specific innate immune recognition mechanisms unique for predisposed individuals that redirect the immune response from tolerance to an allergen-specific maladaptive Th2-polarized response [16].

In this respect, the major birch pollen allergen Bet v 1, a member of the family 10 of plant pathogenesis-related proteins [17], has been studied in great detail. Bet v 1-related proteins share a highly conserved architecture, the Bet v 1-fold, that has been associated with the binding and trafficking of hydrophobic compounds [17, 18]. Among the widely distributed members of the Bet v 1-like superfamily, only Bet v 1 and some homologs from other tree pollens seem to have the unique capacity to sensitize predisposed individuals [17]. Bet v 1 homologs from plant foods which are responsible for the birch pollen-plant food allergy syndrome [19, 20] act as allergens only by cross-reacting with Bet v 1-induced IgE and T-cells [21, 22]. Why only Bet v 1 possesses unique IgE-inducing properties remains unknown. Monocyte-derived dendritic cells (MoDCs) of birch pollen allergic individuals activated by Bet v 1, but not by homologous food allergens, were able to drive a Th2 polarization of the ensuing immune response [23]. Modification of the typical Bet v 1 fold destroyed the protein’s capacity for Th2 polarization resulting in altered T-cell responses [24, 25]. However, the cellular mechanisms that initiate these Th2-biased immune responses are not well understood.

DCs are major players in tuning the fine balance between tolerance induction and active immunity. They are equipped with innate immune receptors, take up and process antigens, and present antigenic peptides in the context of MHC molecules to prime naïve T cells [26]. Thereby, DCs determine whether a protein will be treated as an allergen or an innocuous antigen [6, 7].

Here, we studied the uptake of Bet v 1 and the cross-reactive celery allergen Api g 1 by immature MoDCs (iMoDCs) of allergic and normal donors, and characterized the allergen-triggered intracellular signaling events. Our results demonstrate that DCs of both donor groups discriminate between Bet v 1 and the structurally homologous Api g 1. Bet v 1 targets signaling pathways independent of the Fcε receptor-mediated signaling cascade and promotes a Th2-polarized immune response exclusively in MoDCs of BP allergic donors.

## Methods

### Ethics statement

The study was approved by the Ethics Committee of the Medical University of Vienna (EK Nr: 038/2009). Informed written consent was obtained from all participants.

### Characteristics of patients and ethics statement

Heparinized or EDTA-treated blood was obtained outside the birch pollen (BP) season from BP allergic individuals who had never undergone specific immunotherapy. All donors (3 female, 5 male, mean age 33.7 years) suffered from allergic rhinoconjunctivitis to BP and allergic symptoms after eating BP-related foods (S1 Table). Sensitization to BP was documented by case history, positive skin prick test, and CAP classes ≥ 3 to birch pollen (ImmunoCAP, Phadia, Uppsala, Sweden). Adverse reactions to pollen-related foods were assessed by standardized interviews. Sera of all eight allergic individuals displayed IgE reactivity to Bet v 1.0101. Five patients also displayed IgE reactivity to Api g 1.0101 as determined by ELISA (S1 Table). Seven healthy donors who had no history of reactions to BP or any other allergen source were matched with the allergic group by gender and age. Their sera did not display IgE reactivity to both allergens.

### Purification and characterization of allergens

Bet v 1.0101 and Api g 1.0101 were expressed in *E*. *coli* BL21[DE3] and purified by ion exchange chromatography, gel filtration, and hydrophobic interaction chromatography. Bacterial contaminants such as flagellin, lipoproteins, and S-layer proteins were removed by these purification procedures. Bacterial endotoxins, DNA, and RNA were removed by treating proteins with Endotoxin Removal Beads according to the manufacturer’s protocol (Miltenyi Biotec, Bergisch Gladbach, Germany). The content of endotoxins was determined by the Limulus Amebocyte Lysate assay (Lonza, Walkersville, MD, USA). Peptidoglycan (PGN) and β-glucan content was measured using the Silkworm Larvae Plasma Reagent Set (Wako Pure Chemical Industries, Osaka, Japan). The co-stimulatory effects of trace amounts of LPS or PGN contained in the protein preparations were tested by incubating iMoDCs with 6 pg/ml to 1 μg/ml LPS or 1 ng/ml to 10 μg/ml PGN. Activation of LPS- and PGN-responsive genes such as IL-6, IL-1β, and TNF-α was evaluated by real-time PCR analysis using primers listed in S2 Table.

### Determination of the aggregation state of the allergens

Size exclusion chromatography of aqueous protein solutions was performed using a Superdex S75 10/300 column (GE Healthcare, Little Chalfont, UK). After injection of 75 μl of Bet v 1 or Api g 1 (each 1 mg/ml), proteins were eluted with 50 mM Na-phosphate buffer, pH 7.4, containing 150 mM NaCl and 0.02% (w/v) sodium azide. The column was calibrated for molecular weight determination by injecting the Bio-Rad Gel Filtration Standard (Bio-Rad Laboratories, Hercules, CA, USA) and bovine aprotinin (Sigma-Aldrich, St. Louis, MO, USA). Samples were run in duplicates.

### Fluorescent labeling of Bet v 1 and Api g 1

Allergens were labeled with Alexa dyes (Invitrogen, Carlsbad, CA, USA) according to the manufacturer’s instructions. Briefly, solutions containing 10 mg/ml Alexa Fluor 488 or Alexa Fluor 610 succinimidyl esters dissolved in dry dimethyl sulfoxide were added to the allergens (1 mg/ml in 0.1 M sodium carbonate, pH 8.5) at the recommended dye to protein molar ratio. Incubation was carried out for 1 hour at room temperature in the dark. The conjugates were purified by dialysis against phosphate buffered saline (PBS) overnight at 4°C. The degree of labeling of the conjugates was determined by measuring the absorbance at 495 nm (Alexa 488) or 624 nm (Alexa 610) and 280 nm and was shown to be similar for both proteins. The labeled allergens were stored protected from light at -20°C until use.

### Circular dichroism (CD) spectroscopy

Secondary structures of untreated and labeled proteins were measured by CD spectroscopy at 0.1 mg/ml in 10 mM sodium phosphate, pH 7.5, using a J-810 spectropolarimeter (Jasco, Easten, MD, USA). Far UV spectra between 190 and 260 nm were recorded using a 0.2 cm path length quartz cell (Hellma, Müllheim, Germany). The spectra obtained from three consecutive scans were averaged.

### IgE ELISA and ELISA inhibition

Serum IgE specific for Bet v 1 and Api g 1, both unlabeled or fluorescence labeled, was measured by ELISA as described previously [27]. ELISA inhibition experiments were performed to confirm that labeled Bet v 1 and Api g 1 had retained their full IgE binding capacity. Inhibition of IgE-binding to the unlabeled allergens was measured using individual patients’ sera preincubated with unlabeled or labeled proteins at final concentrations of 1, 10, 100, and 1000 ng/ml.

### Generation and characterization of iMoDCs

iMoDCs were generated by culturing peripheral blood monocytes containing > 90% CD14^+^ cells for 7 days using IL-4 and GM-CSF as described [28]. The purity of the cells was checked by real-time PCR profiling analyzing the mRNA expression levels of cell-type specific markers (HLA-DR/CD40/CD80/CD86 for iMoDCs, CD14 for monocytes, CD19 for B cells, and CD3 for T cells; for primer sequences see S2 Table). In addition, flow cytometry with a FITC anti-human Lineage Cocktail and individual markers such as CD11c-APC, CD3-APC, CD14-FITC, CD19-PE, CD20-APC/Cy7, CD56-PerCP/Cy5.5 (Biolegend, San Diego, CA, USA) was performed. 5x10^3^ events were acquired with a FACSCanto unit (BD Biosciences, San Jose, CA, USA) and analyzed using the FACSDiva software (BD Biosciences).

### Allergen uptake studies

Immature MoDCs were grown at 2x10^5^ cells per well on Lab-Tek glass or Permanox chamber slides (NUNC, Roskilde, Denmark). The cells were stained with Hoechst 33342 (Invitrogen) to label the nuclei before adding Alexa-labeled allergens at 20 μg/ml. Internalization of labeled allergens was followed by live-cell fluorescence imaging using an inverted microscope Axio Observer Z1 equipped with a high resolution AxioCam MRc 5 camera (Carl Zeiss, Jena/Göttingen, Germany). Cells were exposed to labeled allergens for 15 minutes at room temperature to ensure cell surface binding, followed by incubation in allergen-free medium at 37°C and imaging at 15-minute intervals for periods extending up to 4 hours. For uptake competition experiments, unlabeled proteins at final concentrations of 2, 20, and 200 μg/ml were added simultaneously with 20 μg/ml of labeled allergen.

To identify an uptake mechanism for Bet v 1 and Api g 1, cells were preincubated with 5, 25, or 50 μM phenylarsine oxide (PAO, Sigma-Aldrich), 1 μM filipin (Sigma-Aldrich), 200 μM monodansylcadaverine (MDC, Sigma-Aldrich), inhibitors of receptor-mediated endocytosis, caveolae-mediated and clathrin-mediated uptake, respectively, for 30 minutes at 37°C before adding the labeled allergens. Cholera toxin B subunit (CTB, Sigma-Aldrich), and transferrin (Sigma-Aldrich) both conjugated to Alexa Fluor 488, were included as positive controls for caveolae-mediated and clathrin-mediated uptake, respectively.

### Immunoblot analysis of signaling proteins

iMoDCs, plated at 1x10^5^/well in 6-well plates in a final volume of 1 ml, were serum starved for 4 hours and then stimulated in IMDM containing 1% FCS (BioWhittaker) and 20 μg/ml Bet v 1, Api g 1, or a control stimulus for 5, 10, 20, and 30 minutes or left untreated. The control stimulus solution contained 50 ng/ml TNF-α (Strathmann, Hamburg, Germany), 10 ng/ml IL-1β, and 500 U/ml GM-CSF (both PeproTech, Rocky Hill, NJ, USA) and is referred to in the text as maturation-inducing factors (MFs). Cells were lysed in 90 μl Laemmli buffer and the lysates heated for 5 min at 95°C. Protein extracts were separated on 10% SDS gels and transferred to a nitrocellulose membrane (GE Healthcare, Maidstone, UK). Blocking was carried out for 1 hour at room temperature with PBS containing 0.1% Tween 20 (PBST) and 5% skim milk. Primary antibodies (anti-PKCα, anti-pPKCα, anti-Erk1/2 from Santa Cruz Biotechnology, Santa Cruz, CA, USA; anti-pErk1/2, anti-pp38, and anti-IκBα from Cell Signaling Technology, Beverly, MA, USA) were diluted in PBST containing 5% BSA and incubated overnight at 4°C. Bound antibodies were visualized by anti-IgG antibodies conjugated with peroxidase (Sigma-Aldrich) and subsequent chemiluminescence detection (LumiGLO, Cell Signaling Technology).

### Gene expression profiling

iMoDCs were plated at 1x10^6^ cells/well in 6-well plates and treated with 20 μg/ml Bet v 1, 20 μg/ml Api g 1, MFs, or allergens in combination with MFs for 0.5, 1, 2.5, 4, and 24 hours, or were left untreated. To analyze FcεR-mediated gene activation, 3x10^6^ monocytes or 1x10^6^ iMoDCs were incubated with 20 μg/ml Bet v 1, 10 μg/ml polyclonal goat anti-human IgE Fc antibodies (Nordic Immunological Laboratories, Tilburg, Netherlands), or 1 μg/ml human myeloma IgE (Diatec Monoclonals, Oslo, Norway) plus polyclonal goat anti-human IgE.

Total RNA was isolated using the Absolutely RNA Miniprep Kit (Stratagene, La Jolla, CA, USA). One microgram of total RNA was reverse transcribed using the High-Capacity cDNA Reverse Transcription Kit (Applied Biosystems, Foster City, CA, USA) according to the manufacturer’s instructions. Gene expression profiling was performed by real-time PCR analysis on an ABI PRISM 7900HT (Applied Biosystems) as described by Mechtcheriakova et al. [29] in 96-well plate and 384-well array formats.

The Human Immune Array (Applied Biosystems) was used to identify inflammation and immunity-related genes modulated by Bet v 1. cDNAs of selected samples (untreated, 2.5 and 4 hour samples pooled, and 24 hours) from a BP allergic and a healthy donor were loaded onto the array containing 96 genes and processed according to the manufacturer’s recommendations.

Based on the obtained results, primers specific for genes with modulated expression after allergen exposure were used to analyze additional patients’ samples. The early growth response gene family members (EGR-1, -2, and-3) were included as markers of early cell activation. Primers (S2 Table) were designed using the Applied Biosystems Primer Express 2.2 software and validated using a human tissue panel (Clontech) as described [29]. Each PCR reaction was performed in duplicate with SYBR Green-based detection (Applied Biosystems). Data were analyzed by the ΔΔCT method using the SDS 2.1 software (Applied Biosystems). Expression levels of the target genes were normalized to the average expression levels of housekeeping genes (ubiquitin C and elongation factor 1α) and shown relative to unstimulated cells (time point “0”).

### Detection of cell-surface IgE receptors

For staining of surface-bound IgE, 1x10^6^ monocytes or 2x10^5^ iMoDCs per well were grown on Permanox chamber slides (Nunc). Cells were fixed with 3.7% formaldehyde in PBS and incubated for 1 hour at room temperature with polyclonal rabbit anti-human IgE antibodies (Dako, Glostrup, Denmark) diluted in PBS containing 0.5% BSA. Bound antibodies were stained with goat anti-rabbit antibodies labeled with Alexa 568 (Invitrogen) for 45 minutes at room temperature. Nuclei were stained with Hoechst 33342 (Invitrogen). In addition, cells were incubated with 1 μg/ml human myeloma IgE (Diatec Monoclonals) for one hour at 37°C before fixation.

Slides were scanned on a TissueFAXS unit (TissueGnostics, Vienna, Austria), equipped with a pco.pixelfly CCD camera (PCO, Kelheim, Germany) and an EC Plan NeoFluar 20x/0.5 objective (Carl Zeiss). Filter sets were from Chroma TechnologyCorp (DAPI 350/460 nm; FITC/Cy2 470/525 nm; TxRed/Cy5 620/700 nm). Quantitative analysis of positive cells was performed with the TissueQuest 3.0 image analysis software (TissueGnostics, Vienna, Austria). The calculations are based on the recognition of each nucleus (size and staining intensity as major parameters) followed by the analysis of specific staining. Due to the strong inter-patient variability in the background levels and the mean intensity of nuclear staining reflecting the proliferation level of the cells, cut offs were defined separately for each individual patient.

### Statistical analyses

All data were analyzed using GraphPad Prism 6 (La Jolla, CA, USA). Unless otherwise indicated, all values are represented as mean values ± SD. Differences of gene expression between allergic and normal donors as well as between iMoDCs treated Bet v 1 and Api g 1 were analyzed using the repeated measures two-way Anova and the Sidak’s multiple comparison post-test. Expression patterns of individual lineage markers were compared by the multiple t-tests using the Holm-Sidak method. Time-dependent changes in mRNA expression levels after allergen exposure were compared using the non-parametric Friedman’s test combined with Dunn’s multiple comparison test. P values of less than 0.05 were considered statistically significant and denoted as follows: *p < 0.05, **p < 0.01, and ***p < 0.001.

## Results

### Characterization of the allergens

Size exclusion chromatography revealed that recombinant Bet v 1 consisted exclusively of monomers of a molecular weight of 17.5 kDa (S1 Fig, part A). Api g 1 was present as a monomer containing only 3% high molecular weight aggregates > 480 kDa (S1 Fig, part A).

The CD spectra of both labeled allergens showed a mixture of α-helices and β-sheets comparable to that of the unlabeled proteins (S1 Fig, part B). Labeled Bet v 1 and Api g 1 retained their full IgE-binding capacities as shown by competitive IgE ELISA (S1 Fig part C).

The content of LPS was < 3 EU (corresponding to approximately 300 pg) per mg Bet v 1 or Api g 1. Both proteins contained < 0.01 μg PGN per mg. In order to evaluate the impact of these bacterial contaminants, iMoDCs were incubated with increasing concentrations of LPS and PGN and mRNA levels of the NFκB-responsive genes IL-1β, IL-6, and TNF-α were determined. We observed an increase of mRNA transcript levels of these genes with LPS concentrations starting at 60 pg/ml (4-fold for IL-1β; 12-fold for IL-6). Upregulation of IL-6 and TNF-α mRNA levels by about 111- and 14-fold, respectively, was detected following PGN treatment at 0.1 μg/ml. The amounts of LPS and PGN showing these effects were approximately 10- to 20-fold higher than those present in the recombinant protein solutions used in our study.

### Purity of MoDC preparations

Flow cytometry analysis (S3 Table) showed that the average purity of the MoDC preparations was 89% ± 7% for the BP allergic group and 91% ± 8% for the normal donor group (n = 7 for each group). Cell preparations contained < 9% B cells, < 2% T cells, < 0.6% NK cells, and < 0.6% monocytes (S3 Table, part B). There were no significant variations in the purity of iMoDCs obtained from BP allergic and normal donors.

### Kinetics of the uptake of labeled allergens by iMoDCs

**Fig 1:**
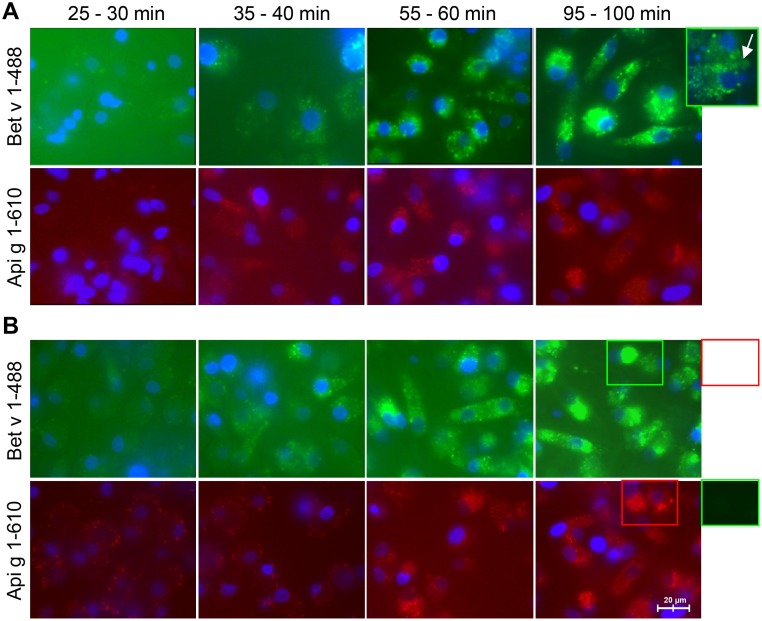
Kinetics of allergen uptake into iMoDCs of BP allergic and normal donors. Internalization of labeled Bet v 1 (Bet v 1–488) and labeled Api g 1 (Api g 1–610) by iMoDCs of allergic (A) and normal donors (B) was followed by live-cell fluorescence imaging. Results are representative of independent experiments of three different donors for each group (shown for donors AD1 and ND3). Exocytosis of fluorescent markers (panel A) and the absence of spillover of fluorescence (panel B) are depicted by framed display details.

We used iMoDCs from three BP allergic and three healthy donors. The labeled allergens were first bound to the cell membrane and then taken up by iMoDCs in a dose- and time-dependent manner (Fig. 1). Protein binding to the cell surface occurred within 10–30 minutes after allergen exposure. Internalization was observed after 35–40 minutes and was visible as a punctuated pattern in the intracellular space. The uptake reached a plateau at 95–100 minutes. Subsequently, fluorescent markers were released by exocytosis resulting in an increased signal in the extracellular space (Fig. 1A, insert). No significant differences were detected for the kinetics of binding and uptake of labeled allergens by iMoDCs of allergic (Fig. 1A) or normal donors (Fig. 1B).

### Bet v 1 and Api g 1 compete for binding to iMoDCs

**Fig 2:**
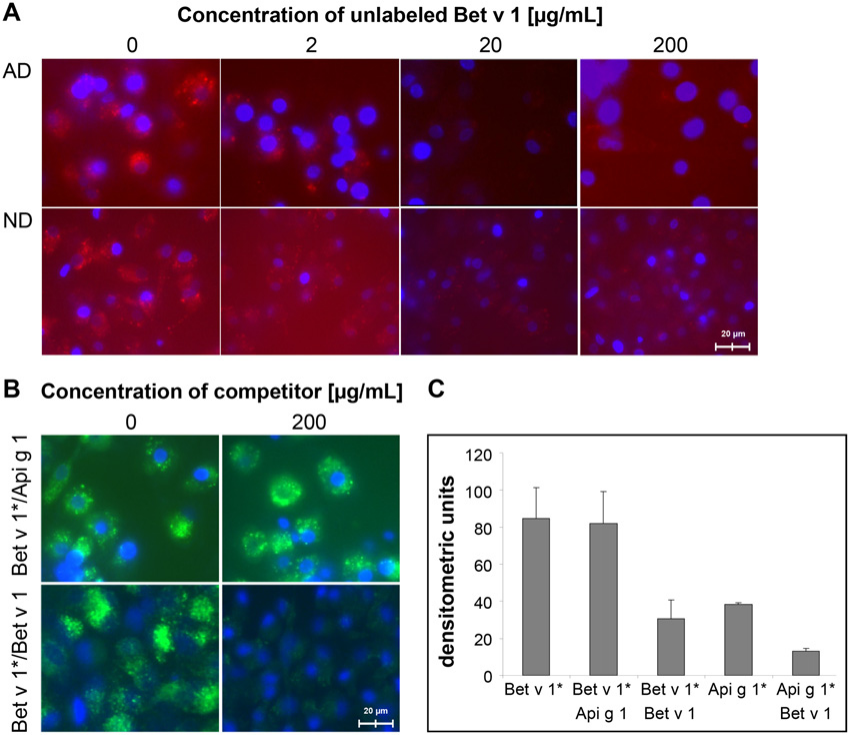
Competitive binding of allergens to iMoDCs of BP allergic (AD) and normal donors (ND). (A) Uptake of labeled Api g 1 (20 μg/ml) in the presence of unlabeled Bet v 1 (shown for donors AD3 and ND3). (B) Cross-competition of labeled Bet v 1 with Api g 1 (Bet v 1*/Api g 1) and self-competition of Bet v 1 (Bet v 1*/Bet v 1) using 20μg/ml of labeled allergen and 200 μg/ml of competitor (donor AD3). Results in (A) and (B) show uptakes after 45 minutes of chase and are representative of four independent experiments with similar outcomes for both donor groups. (C) Densitometric quantification of the competition assays measuring a 1 mm^2^ area.

iMoDCs were incubated with a fixed amount of fluorescence labeled allergen and increasing amounts of unlabeled allergen as competitor. We observed a dose-dependent competition between the uptake of labeled Api g 1 and unlabeled Bet v 1 (Fig. 2A). Equimolar amounts of Bet v 1 prevented the binding of Api g 1 to the cell surface of iMoDCs of both BP allergic and normal donors. In this assay, a 60–70% inhibition of Api g 1-uptake by unlabeled Bet v 1 was observed (Fig. 2C). In turn, Api g 1 delayed the intracellular accumulation of Bet v 1 only at early time points but no significant competition even at a ten-fold higher concentration of Api g 1 was observed (Fig. 2B). In self-competition experiments, unlabeled Bet v 1 at 200 μg/ml abrogated the uptake of labeled Bet v 1 by up to 80% (Fig. 2B and 2C).

### Uptake of Bet v 1-homologous proteins is sensitive to inhibition of endocytic pathways

**Fig 3:**
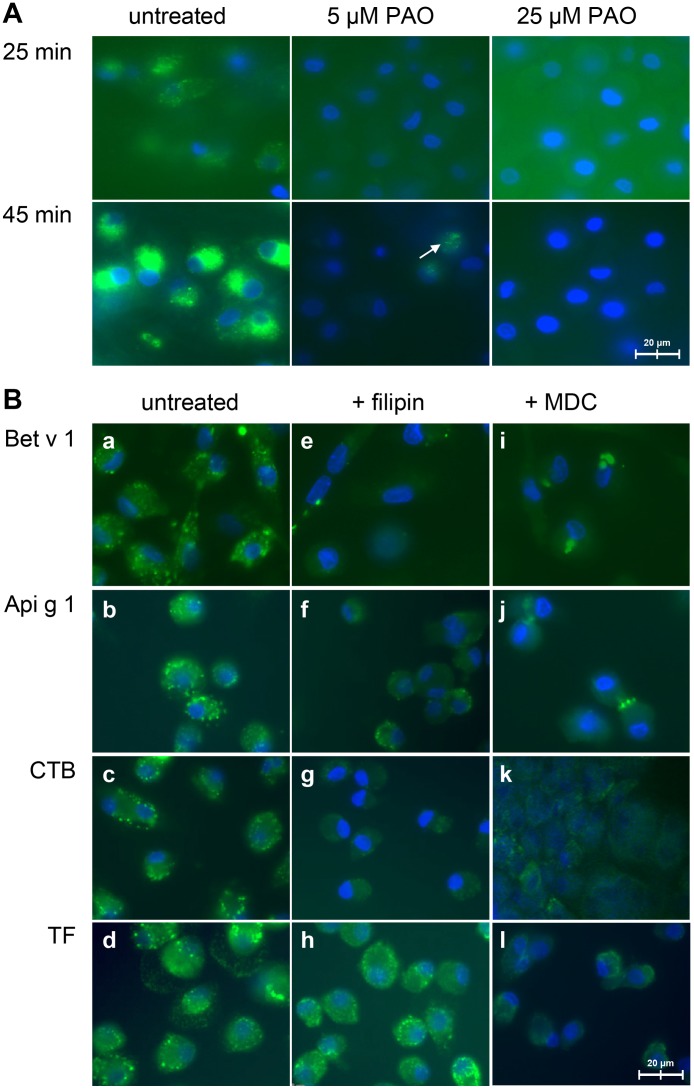
Uptake of Bet v 1 and Api g 1 in the presence of pharmacological inhibitors. (A) Uptake of labeled Bet v 1 (20 μg/mL) by iMoDCs pre-treated with PAO. Images are representative of three independent experiments using cells of BP allergic donors. An arrow indicates residual Bet v 1 uptake after PAO treatment at 5 μM. (B) Effects of filipin (1 μM) and MDC (200 μM) on the uptake of Bet v 1, Api g 1, CTB, and transferrin (TF). Images are representative of four independent experiments using cells of BP allergic and normal donors (n = 2 for both groups).

Pre-incubation of iMoDCs of allergic donors with PAO, an inhibitor of the internalization of cell surface receptors, resulted in a concentration-dependent inhibition of the endocytosis of Bet v 1 (Fig. 3A). At 5 μM PAO, trace amounts of Bet v 1 were detected within the cells while at 25 μM PAO no signal was observed (Fig. 3A, 45 minutes). Filipin, an inhibitor of caveolae-mediated endocytosis, inhibited the uptake of CTB (Fig. 3B, g) but not of transferrin (Fig. 3B, h) and strongly reduced the amount of internalized Bet v 1 and Api g 1 (Fig. 3B, e and f). Inhibition of the clathrin-mediated pathway by MDC strongly reduced the uptake of transferrin (Fig. 3B, l). We observed that fluorescent Bet v 1 and Api g 1 formed larger extracellular and intracellular aggregates in the presence of MDC resulting in a reduced uptake (Fig. 3B, i and j). However, the internalization of CTB was as well reduced upon pretreatment with MDC (Fig. 3B, k) suggesting that MDC also interfered with caveolae-mediated uptake.

### Differential activation of MAP kinase pathways upon allergen stimulation

**Fig 4:**
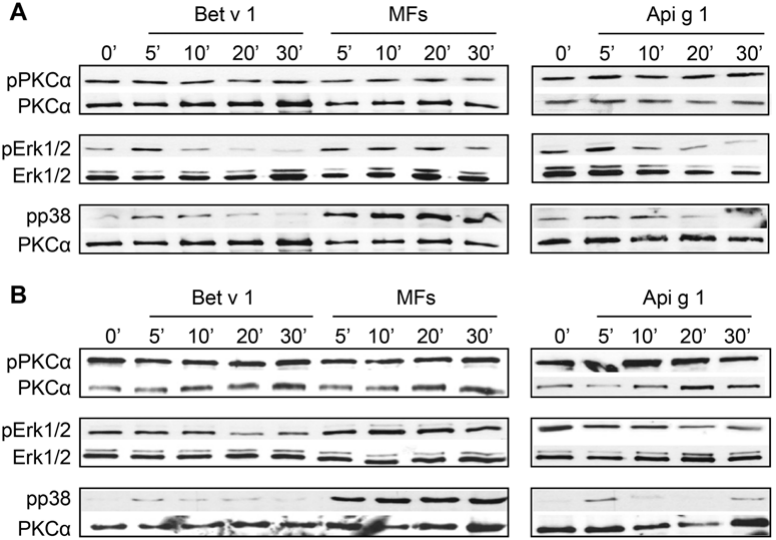
Phosphorylation of MAP kinases after activation by allergens. Cells of BP allergic (A) and normal donors (B) were stimulated with the allergens (each 20 μg/ml) or control stimulus (MFs) for the indicated time points and then lysed. Cell extracts were analyzed by Western blot using antibodies specific for the phosphorylated forms of PKCα, Erk1/2, and p38 MAPKs. Sample amounts were determined with antibodies to Erk1/2 and PKCα. The blots shown are representative of independent experiments with cells of three donors for each group yielding similar results (shown for donors AD2 and ND1).

Both Bet v 1 and Api g 1 triggered transient Erk1/2 phosphorylation in iMoDCs of allergic donors and phosphorylation of p38 in both donor groups (Fig. 4). No activation of JNK was detected. Phosphorylation of PKCα, one of the potential upstream kinases of Erk1/2, was not increased upon stimulation with allergens in both donor groups (Fig. 4).

### Bet v 1 induces a Th2-dominant transcriptional program only in iMoDCs of BP allergic donors

An initial screen of Bet v 1-induced expression of inflammation- and immunity-related genes was performed using a pre-designed 96-gene array. Six genes were differentially expressed after allergen stimulation: IL-3, IL-4, IL-5, IL-13, CXCL10 and CXCL11 (S4 Table). The time course of the regulation of these genes by the allergens was then studied. Additionally, EGR family members as markers of early cell activation and the pro-inflammatory cytokines IL-1β and IL-6 as markers of NFκB activation were included.

**Fig 5:**
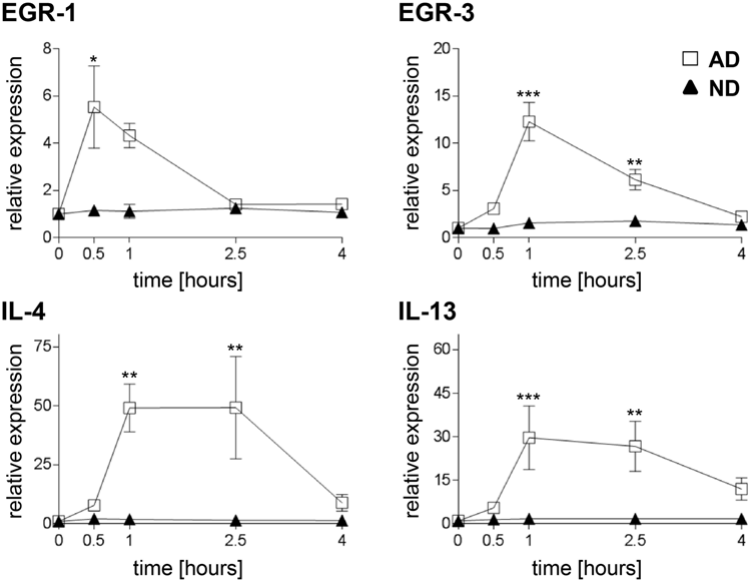
Gene expression of MoDCs induced by Bet v 1. Cells of BP allergic (squares) and normal donors (triangles) were either sham-treated or treated with Bet v 1 for the indicated times. mRNA levels of EGR-1 and-3 and the Th2 cytokines IL-4 and IL-13 were analyzed by real-time PCR. Expression levels, normalized to the average of housekeeping genes, are shown relative to non-stimulated cells. Data are presented as mean values ± SEM (n = 7 in both groups) performed in duplicates. Significance is indicated by asterisks (* p < 0.05; ** p < 0.01; *** p < 0.001).

**Fig 6:**
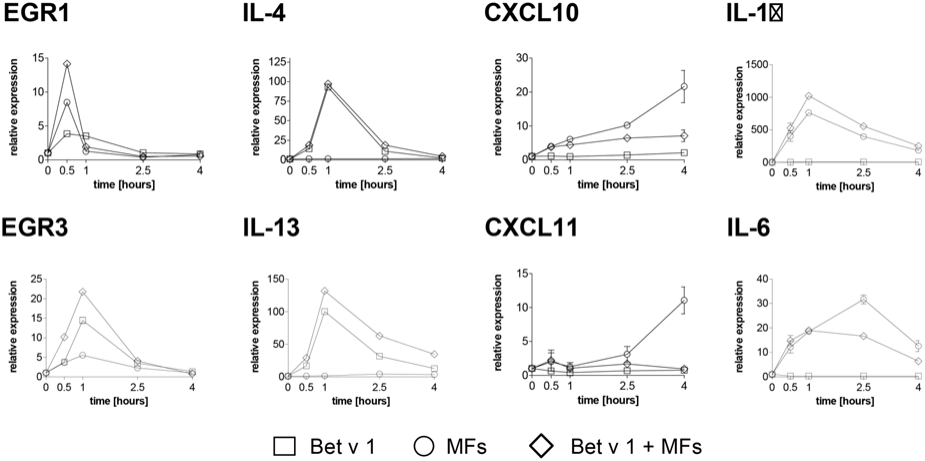
Gene expression induced by Bet v 1 and MFs in iMoDCs of BP allergic donors. Cells were sham-treated or treated with Bet v 1 (squares), control stimulus (MF, circles), or a combination of Bet v 1 and control stimulus (diamonds) for indicated time periods. mRNA levels of EGR-1 and-3, Th2 cytokines IL-4 and IL-13, Th1 chemokines CXCL10 and 11, and pro-inflammatory cytokines IL-1β and IL-6 were analyzed by real-time PCR. Expression levels, normalized to the average of housekeeping genes, are shown relative to unstimulated cells. Data for donor AD2 are shown, which are representative of independent experiments with mRNA from cells of three donors each performed in duplicates.

Bet v 1 stimulation of iMoDCs from BP allergic donors resulted in a transient upregulation of EGR-1 mRNA with maximum expression levels at 0.5 hours after stimulation (p < 0.05; Fig. 5). EGR-3 mRNA showed a maximal upregulation at 1 hour post stimulation with an up to 18-fold induction (mean fold induction of 12.3 ± 4.9, p < 0.001; Fig. 5). An additive effect on the increase of mRNA levels was observed after combined administration of Bet v 1 and MFs (Fig. 6). EGR-2 mRNA expression levels were unaffected by the treatment with Bet v 1 or MFs.

In iMoDCs from allergic donors only, mRNA levels of the Th2 cytokines IL-4 and IL-13 showed a sustained upregulation by Bet v 1 (Fig. 5; p < 0.01 and p < 0.001, respectively). MFs did not influence the expression of these cytokines (Fig. 6). No significant upregulation of IL-3 and IL-5 after allergen stimulation was observed. Api g 1 induced a similar gene expression profile but with considerably lower relative expression levels compared with Bet v 1 (S2 Fig).

mRNA levels of the Th1-related chemokines CXCL10 and CXCL11 as well as the pro-inflammatory cytokines IL-1β and IL-6 were upregulated by the MF stimulus but not by Bet v 1 in allergic donors. Furthermore, their expression levels were reduced 2- to 3-fold when MFs were applied in combination with Bet v 1 (Fig. 6). Similarly, iMoDCs of healthy donors exhibited an upregulation of CXCL10 and CXCL11 mRNA following stimulation by MFs. However, the expression of these chemokines was not downregulated by addition of Bet v 1 (S3 Fig).

### IgE is not required for binding of Bet v 1 and Api g 1 to iMoDCs

We compared mRNA levels of the IgE receptors FcεRI and FcεRII/CD23 of iMoDCs of BP allergic and normal donors. iMoDCs of both donor groups displayed comparable mRNA levels for CD23 and the FcεRI subunits alpha (FcεRIα) and gamma (FcεRIγ) but lacked transcripts for the beta subunit (S4 Fig).

**Fig 7:**
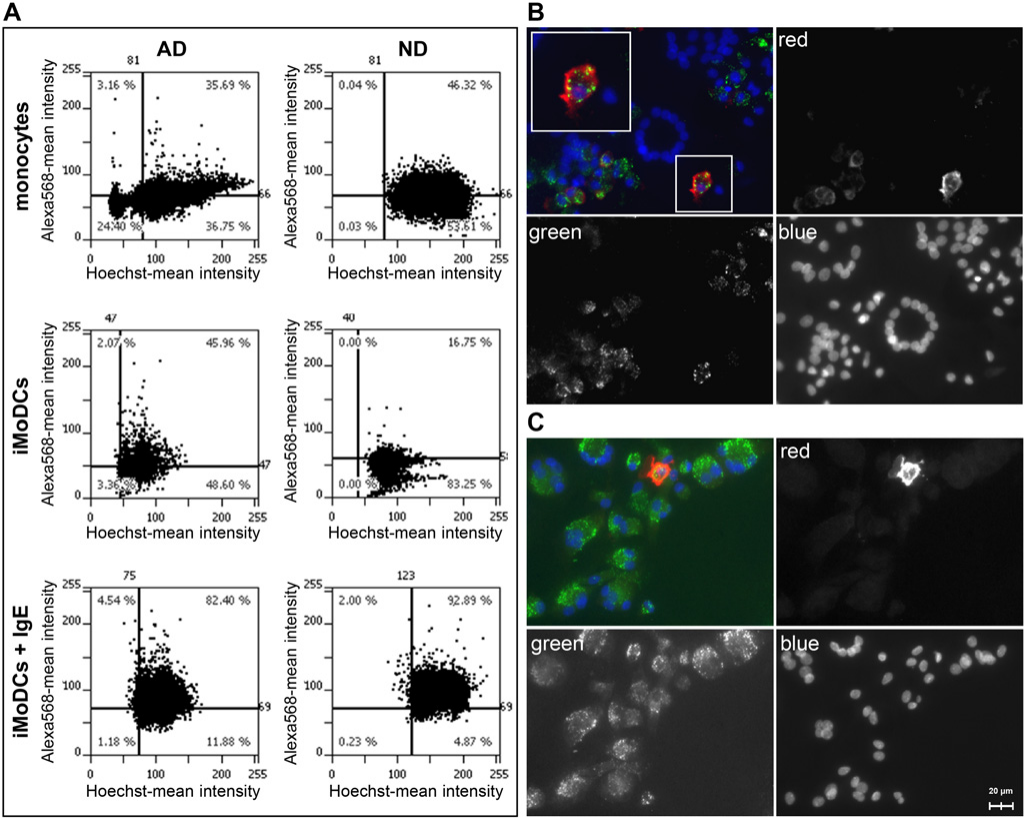
Binding of IgE and Bet v 1 to monocytes and iMoDCs of BP allergic (AD) and normal donors (ND). Cells were grown in chamber slides and stained with Hoechst33342 to label the nuclei. Surface-bound IgE was detected with primary antibodies against IgE and Alexa Fluor 568-labeled secondary antibodies. (A) Quantification of surface-bound IgE and IgE receptors using a TissueFAXS microscopy system. FcεRI surface expression was determined indirectly by loading iMoDCs additionally with 1μg/ml human myeloma IgE (iMoDCs + IgE). Results represent data typical for two independent experiments for each donor group (shown for donors AD2 and ND2). Percentage of IgE-positive cells is indicated in the upper right corner of the scattergram. (B) Fluorescence microscopy of iMODCs of BP allergic and (C) normal donors. Cells were incubated with Alexa 488-labeled Bet v 1 (green) and Alexa Fluor 568-labeled anti-IgE antibodies (red). The magnified insert in (B) shows an individual cell with non-overlapping staining of Bet v 1 and IgE.

Surface expression of IgE receptors was determined indirectly by staining for surface-bound IgE (Fig. 7). Thirty-five to 46% of the monocytes from BP allergic and normal donors showed staining for surface-bound IgE (Fig. 7A, upper graphs). After differentiation, lower percentages of IgE positive MoDCs (range 16–20%) were determined for the normal donors while allergic donors still displayed 35–45% positive cells (Fig. 7A, middle graphs). Addition of saturating amounts of myeloma IgE restored receptor occupancy resulting in up to 92% IgE-positive iMoDCs in both donor groups (Fig. 7A, lower graphs). Bet v 1-uptake was equally efficient in IgE-positive and-negative cells with non-overlapping fluorescence patterns for Bet v 1 (labeled in green) and IgE (visualized by anti-IgE in red) in both donor groups (Fig. 7B and C).

### Bet v 1 and Fcε receptor cross-linking induce the expression of different genes in iMoDCs of BP allergic donors

**Fig 8:**
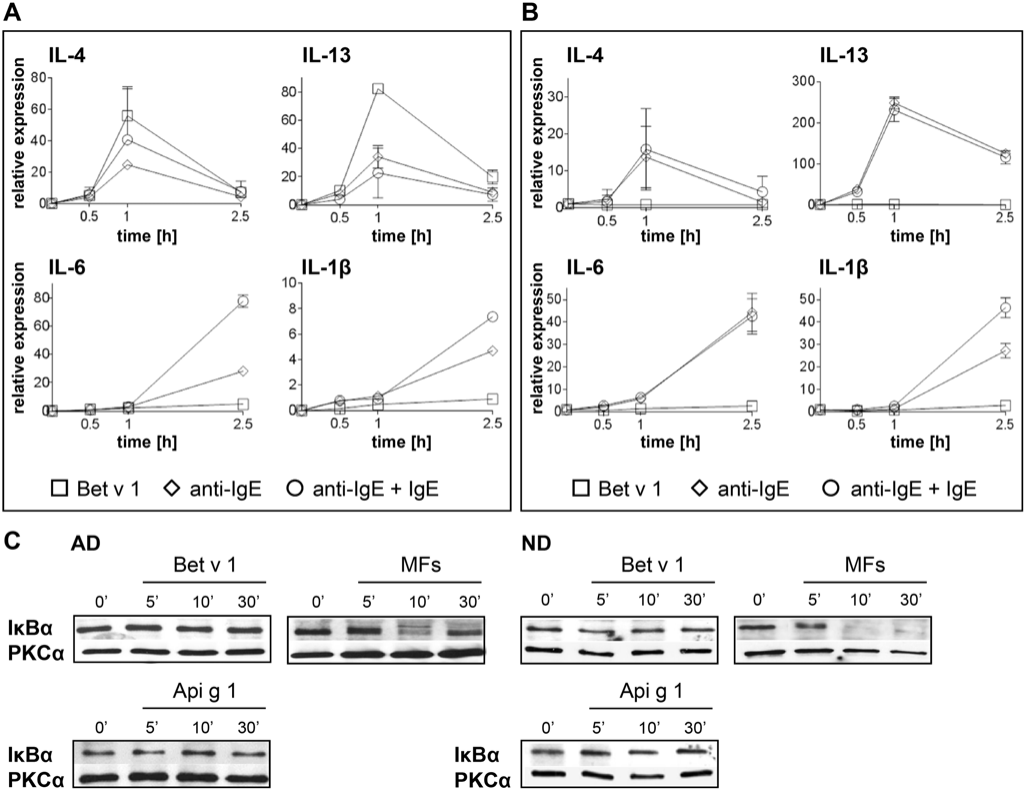
Gene expression of iMoDCs after Bet v 1 stimulation or cross-linking of cell-bound IgE. iMoDCs of BP allergic (A) and normal donors (B) were stimulated with Bet v 1 (squares), anti-IgE (diamonds), or loaded with IgE before anti-IgE treatment (IgE+anti-IgE, circles). mRNA levels of IL-4, IL-13 and the NFκB related genes IL-1β and IL-6 were analyzed by real-time PCR. Error bars indicate SD. Results are representative of two independent experiments for each donor group (shown for donors AD1 and ND1). (C) Activation of NFκB was evaluated by detection of its inhibitory protein IκBα. The Western blots shown are representative of independent experiments for three donors of each group yielding similar results (shown for donors AD2 and ND2).

We determined the induction of IL-1β and IL-6 as markers for NFκB activation by FcεRI/CD23 cross-linking. Upregulation of IL-4 and IL-13 was taken as an indication of Bet v 1-induced gene expression. Anti-IgE-induced Fcε receptor cross-linking triggered an upregulation of IL-1β and IL-6 in iMoDCs of allergic and normal donors (Fig. 8A and B). Additionally, a moderate upregulation of Th2 cytokines was detected. In contrast, Bet v 1 stimulation induced an upregulation of IL-4 and IL-13 but not of IL-1β and IL-6 exclusively in iMoDCs of BP allergic donors, with values higher than those after anti-IgE treatment (Fig. 8A).

The activation of NFκB was additionally evaluated by determining degradation of IκBα. Treatment with the MF cocktail, which was used as a positive control, led to a rapid decrease of IκBα levels after 10 minutes of stimulation (Fig. 8C). In contrast, IκBα expression remained unchanged following stimulation with Bet v 1 or Api g 1 (Fig. 8C). Thus, in line with the real-time PCR-based gene expression data, Bet v 1 stimulation did not activate an NFκB-dependent signaling cascade.

## Discussion

The ability of allergens to initiate Th2-polarized immune responses appears to result largely from the direct engagement of innate immune pathways [2, 30]. This polarization is only induced by a very limited number of proteins [31]. Moreover, not every individual exposed to these proteins develops allergy indicating differences in signal processing between immune cells predisposed individuals and the non-allergic population. Hence, the goal of this study was to characterize the events after allergen encounter including uptake, induction of signal transduction pathways, and gene regulation in antigen presenting cells. We based this study on the comparative analysis of the interactions of iMoDCs from BP allergic and normal donors with the major BP allergen Bet v 1 and the structurally homologous but weak allergen Api g 1 from celery.

Here, we show that iMoDCs of BP allergic and normal donors are able to internalize Bet v 1 and Api g 1 with similar kinetics (Fig. 1). However, cells incubated with both allergens at the same time preferentially internalize Bet v 1. DCs appear to be able to discriminate between these two structurally related allergens as shown by concentration-dependent competitive binding (Fig. 2). This suggests that a Bet v 1-specific receptor-mediated uptake exists in addition to the uptake by macropinocytosis described previously [32]. Macropinocytosis would not result in the preferred uptake of a certain protein [33]. Alterations in structural or immunological properties of the allergens caused by protein aggregation or the presence of the fluorescent label were excluded as the reason for the observed competition (S1 Fig).

We used pharmacological inhibitors to interfere with different internalization pathways. Bet v 1-uptake was sensitive to PAO (Fig. 3A) suggesting involvement of receptor-mediated endocytosis [34]. Thus, to determine the route of Bet v 1-internalization more precisely, we inhibited the clathrin-mediated and caveolae-mediated pathways using MDC and filipin [34], respectively. The uptake of Bet v 1 and Api g 1 was unaffected by MDC but strongly reduced after treatment with filipin (Fig. 3B). These results suggest that, in addition to macropinocytosis, Bet v 1 and its structural homolog Api g 1 are taken up in a receptor-dependent manner via caveolae-mediated endocytosis.

Biochemical and structural data indicate that Bet v 1 is able to accommodate a wide range of amphiphilic and lipid ligands via its hydrophobic cavity [18, 35, 36], including ceramide, sphingomyelin, and glycolipids which are integral parts of caveolae and lipid rafts [37]. The ability of Bet v 1 to interact with lipids might be involved in its uptake. Notably, Renkonen et al. [38] and Joenvaara et al. [39] showed the caveolae-mediated passage of Bet v 1 through conjunctival and nasal epithelia of BP allergic patients. Caveolae are a special type of lipid rafts which represent platforms that regulate cell signaling and immune responses by placing receptors and signaling molecules in spatial vicinity [37]. This prompted us to ask whether the induction of signaling pathways and the expression of immune-relevant genes following Bet v 1- and Api g 1-exposure would be different in iMoDCs of BP allergic and normal donors.

We showed that Bet v 1 was able to induce Erk1/2 and p38 MAPK activation in BP allergics but only a slight p38 activation in normal donors (Fig. 4). Modulation of MAP kinases in DCs following exposure to classical Th1 or Th2 stimuli is thought to determine their polarizing capacity [40–42]. While Erk1/2 phosphorylation has been related to Th2 responses, negatively regulating IL-12 production by DCs, p38 phosphorylation is known to favor the activation of Th1-promoting DCs [40, 43, 44]. Thus, our data indicate that Bet v 1 favors the activation of a Th2 program only in DCs of BP allergic individuals. Although Bet v 1 and Api g 1 showed similar activation of MAPK in iMoDCs of BP allergic donors (Fig. 4), already subtle differences in the Erk/p38 ratio might be decisive for the expression levels of cytokines as shown by van Riet et al. [43]. This is also consistent with a previous report showing that native or deglycosylated peanut allergen induced different Erk/p38 ratios in MoDCs and altered up-regulation of cell surface markers and T cell polarization [44]. Nevertheless, contribution of other signaling pathways induced by Bet v 1 exclusively or to a stronger extent than by Api g 1 cannot be excluded and needs further studies to be resolved.

Although the activation of Erk1/2 by Bet v 1 in iMoDCs of BP allergic donors was transient in comparison to the one triggered by the cytokine cocktail, the MAPK/Erk downstream target genes such as EGR family members [45] were up-regulated (Fig. 5). Therefore, despite its transient nature, the Erk1/2 activation seems to have biological relevance. We further showed that Bet v 1 was able to trigger Th2 signature cytokine genes only in iMoDCs of BP allergic donors (Fig. 5). The Th2-promoting capacity of Bet v 1 was further supported by its ability to down-regulate the MF-induced chemokines CXCL10 and CXCL11 (Fig. 6), which are linked to Th1 immunity [46]. Rapid upregulation of EGR-1 or EGR-3, as observed in our study, can be induced by various extracellular stimuli. These transcription factors might serve as potent activators of inflammatory processes through the transcriptional control of the Th2 cytokines IL-4 and IL-13 [47, 48]. In agreement with our gene expression data, IL-13 transcription by DCs of allergic donors in response to Bet v 1 seems to be a critical step towards differentiation of potentially Th2-driving DCs [49]. Although IL-4 is believed to be indispensable for the induction and maintenance of Th2 responses [50, 51], DCs are generally not considered to be a source of IL-4 [26]. Yet, there are reports in the literature that demonstrate secretion of IL-4 by both human and mouse dendritic cells [52–54].

In contrast to allergic donors, normal donor-derived iMoDCs were mostly unresponsive to allergen stimulation (Fig. 5). It is conceivable that allergen presentation by these cells to naïve T cells results in tolerance. These data corroborate our previous finding that Bet v 1-loaded MoDCs of BP allergic, but not of normal donors, were able to induce Th2 cytokines in autologous T helper cells [23].

As Fcε receptor-bound IgE was found to be present on the cells in our study (Fig. 7A), it cannot be excluded that Bet v 1-specific IgE might contribute to the differences in allergen recognition and signaling. However, the competitive binding of Bet v 1 and Api g 1 to cells of both donor groups (Fig. 2) as well as the lack of Bet v 1-colocalization with surface bound IgE (Fig. 7B) suggest that the observed effects do not simply reflect Fcε receptor engagement via Bet v 1-specific IgE.

In human monocytes and MoDCs, the cross-linking of surface IgE receptors, both FcεRI and FcεRII/CD23, leads to the degradation of the NFκB inhibitory molecule IκB-α, subsequent nuclear translocation of the NFκB subunits, and activation of pro-inflammatory cytokines [55, 56]. In line with that, we showed that FcεR cross-linking via IgE/anti-IgE upregulated the NFκB-dependent genes IL-1β and IL-6 (Fig. 8). However, NFκB was not involved in Bet v 1-induced signaling and gene expression in iMoDCs of BP allergic and normal donors suggesting the induction of an FcεR-independent pathway. In support of our results, studies performed on MoDCs of grass pollen allergic patients showed the expression of NFκB-dependent cytokines only in cells exposed to the major grass pollen allergen Phl p 5 in complex with Phl p 5-specific IgE but not by Phl p 5 alone [57]. In addition to the well-known uptake of allergens via cell-bound IgE [58] our data indicate an FcεR-independent internalization of Bet v 1 by an unknown receptor located in caveolae resulting in an upregulation of IL-4 and IL-13 mRNA (Figs. 5 and 6).

In summary, our data illustrate different signal transduction events in DCs of allergic and non-allergic donors despite similar uptake kinetics and mechanisms. Further, our experiments showed that DCs distinguish between Bet v 1 and its structural homologue Api g 1. One of the questions which is still open and will be addressed in follow-up studies is whether the predisposition for sensitization to Bet v 1 is specific for birch pollen allergic patients or exists in all atopic patients. Further characterization of signaling pathways and gene expression signatures that underlie the regulation of allergic and healthy donor-derived DC activation by allergens will be one of the next steps towards understanding the pathogenesis of allergic sensitization.

## Supporting Information

S1 Table Characteristics of birch pollen allergic patients. (PDF)

S2 Table Primer sequences used for real-time PCR analyses. NCBI accession number, gene symbol and synonym, sequences of forward (F) and reverse (R) primers.
(PDF)

S3 Table Expression pattern of lineage markers in iMoDCs of BP allergic and normal donors assayed by flow cytometry. The values are mean ± standard deviation (SD) of 7 independent donors within each group.
(PDF)

S4 Table Differentially expressed genes after allergen stimulation of cells from a BP allergic (A) and a normal donor (B). cDNA was synthesized from mRNA isolated 2.5 h and 4 h after allergen stimulation, pooled and analyzed for expression of 96 genes using the Human Immune Array. Genes whose expression changed by at least 3-fold compared with the baseline levels of unstimulated cells (Bet v 1 vs. unstimulated) or MFs (Bet v 1 + MFs vs. MFs) are shown in bold.
(PDF)

S1 Fig Physico-chemical characterization and IgE binding ability of unlabeled and labeled allergens. (A) Size exclusion chromatography of Bet v 1 and Api g 1. Molecular weight standards are indicated by filled diamonds. (B) CD spectra of unlabeled allergens (black lines) and Alexa-labeled allergens (grey lines) at pH 7.5. (C) Inhibition of IgE binding to Bet v 1 and Api g 1 by unlabeled (black) or labeled Bet v 1 and Api g 1 (grey). Results are represented as mean values and standard deviation of three patients’ sera.
(PDF)

S2 Fig Gene expression induced by Bet v 1 and Api g 1 in iMoDCs of BP allergic donors. Cells were sham treated or treated with Bet v 1 (squares) or Api g 1 (triangles) for indicated time periods. mRNA levels of EGR3 and the Th2 cytokines IL-4 and IL-13 were analyzed by real-time PCR. Expression levels, normalized to the average of housekeeping genes, are shown relative to unstimulated cells. Mean values and SD of duplicate experiments with cells of two donors are shown.
(PDF)

S3 Fig Expression of CXCL10 and CXCL11 in iMoDCs of normal donors. Cells were sham treated or treated with Bet v 1 (squares), a control stimulus (MFs; circles), or a combination of allergen and control stimulus (diamonds) for the indicated time periods. mRNA levels of the Th1 chemokines CXCL10 and 11 were analyzed by real-time PCR. Expression levels, normalized to the average of housekeeping genes, are shown relative to unstimulated cells. Data are presented as mean values ± SD from independent experiments using cells from four different normal donors each performed in duplicates.
(PDF)

S4 Fig Expression of Fcε receptors in iMoDCs. mRNA of unstimulated iMoDCs of BP allergic (AD) and healthy donors (ND) was analyzed by real-time PCR for the expression of FcεRIα, FcεRIβ, FcεRIγ, and CD23. Expression levels were normalized to the average of housekeeping genes. Values are displayed as mean values ± SD of four donors per group.
(PDF)
