# Supplementary material for: The Major Birch Pollen Allergen Bet v 1 Induces Different Responses in Dendritic Cells of Birch Pollen Allergic and Healthy Individuals
Source: PLoS One. 2015 Jan 30;10(1):e0117904. doi: 10.1371/journal.pone.0117904 (PMC4311984; doi:10.1371/journal.pone.0117904)
Supplement: S1 Table — (PDF) [file pone.0117904.s001.pdf]

S1 Table. Characteristics of birch pollen allergic patients.

Patient	Sex	Age [years]	Total IgE [kU/L]	Allergen-specific IgE			Symptoms [a]		Sensitizations to plant foods [b]		Sensitization to other aeallergens [c]	
				Birch [CAP class]	Bet v 1.0101 [ELISA $OD_{405\ nm}$]	Api g 1.0101 [ELISA $OD_{405\ nm}$]	Birch pollen	Plant foods	sIgE [CAP class]	Skin prick test	sIgE [CAP class]	Skin prick test
AD1	m	28	4155	5	1.4	0.1	RC	OAS, AD	Ap=3; Am=1; Pe=3; Pn=1; Sy=1	Ap, Ca	D=1; G=4; H=2; Hz=2; M=1	
AD2	m	40	393	3	0.4	0.1	RC	OAS	Ce=1; Pe=1; Pn=1	Ap, Ca, Ce	G=2; Hz=1; O=1	
AD3	f	30	38	3	0.7	0.0	A, RC	OAS	Sy=2	Ap, Ce, Pe	Al=3; G=3	
AD4	m	27	47	3	1.0	0.0	A, RC	OAS			G=4; Hz=3; Ra=2	Al, C, D, H, Mo, Mu, Ra, Ry
AD5	m	40	444	5	3.8	0.2	RC	OAS			C=3; G=4; Ry=4; M=3	
AD6	f	28	155	4	2.7	0.1	RC	OAS		Ho	G=2	G
AD7	m	49	250	4	2.1	0.1	RC	OAS	Ap=2; Hn=2		G=2	
AD8	f	32	NA	4	2.7	0.0	RC	OAS	Pn=1		G=2; Ra=2; M=1; Al=2	

[a] A = asthma; AD = atopic dermatitis; OAS = oral allergy syndrome; RC = rhinoconjunctivitis

[b] Am = Almond; Ap = apple; Ca = carrot; Ce = celery; Hn = hazelnut; Ho = honey; Pe = peach; Pn = peanut; Sy = soy

[c] Al = *Alternaria*; C = cat; D = dog; G = grass pollen; H = horse; Hz = hazel pollen; M = house dust mites; Mu = mugwort pollen; Mo = mold; O = olive pollen; Ra = ragweed pollen; Ry = rye pollen

NA = Not available
