# Supplementary material for: The Major Birch Pollen Allergen Bet v 1 Induces Different Responses in Dendritic Cells of Birch Pollen Allergic and Healthy Individuals
Source: PLoS One. 2015 Jan 30;10(1):e0117904. doi: 10.1371/journal.pone.0117904 (PMC4311984; doi:10.1371/journal.pone.0117904)
Supplement: S2 Table — NCBI accession number, gene symbol and synonym, sequences of forward (F) and reverse (R) primers. (PDF) [file pone.0117904.s002.pdf]

S2 Table. Primer sequences used for real-time PCR analysis. NCBI accession number, gene symbol and synonym, sequences of forward (F) and reverse (R) primers.

No	Symbol	Synonym	Acc. no.	Primer sequences
**Bet v 1 regulated genes from the gene signature array**				
1	IL3		NM_000588	F: TTGCCTTTGCTGGACTTCAA<br>R: TTGAATGCCTCCAGGTTTGG
2	IL4		NM_172348	F: CGACTGCACAGCAGTTCCA<br>R: CTCTGGTTGGCTTCCTTCACA
3	IL5	TRF	NM_000879	F: TGCTGATAGCCAATGAGACTCTGA<br>R: AGTGTGCCTATTCCCTGAAAGATT
4	IL13	ALRH<br>BHR1	NM_002188	F: ACCTGACAGCTGGCATGTACTG<br>R: TGGGTCTTCTCGATGGCACT
5	CXCL10	INP10<br>SCYB10	NM_001565	F: AAGCAGTTAGCAAGGAAAGGTCTAA<br>R: AGCCTCTGTGTGGTCCATCC
6	CXCL11	ITAC<br>SCYB11<br>SCYB9B	NM_005409	F: GTGTGCTACAGTTGTTCAAGGCTTC<br>R: TTACCCCAGGGCCTATGCAA
**Markers of early cell activation**				
7	EGR-1	KROX24<br>NGFIA<br>ZIF268	NM_001964	F: TGAACGCAAGAGGCATACCA<br>R: AAGCAGGGGGAACAGAGGA
8	EGR-2	KROX20	NM_000399	F: GCAAATGATGACCGCCAAG<br>R: GGATATGGGAGATCCAACGAC
9	EGR-3	PILOT	NM_004430	F: CAATCTGTACCCCGAGGAG<br>R: TGGTCAGACCGATGTCCATTAC
**Genes used to study Fcε receptor activation**				
10	IL1β	IL1F2	NM_000576	F: GTACCTGAGCTCGCCAGTGA<br>R: TCGGAGATTCGTAGCTGGATG
11	IL6	IFNB2	NM_000600	F: GTACATCCTCGACGGCATCTC<br>R: GGCAAGTCTCCTCATTGAATC
12	TNFα	TNFSF2	NM_000594	F: ATGTTGTAGCAAACCCTCAAGCT<br>R: TTGGCCAGGAGGGCATT
**Fcε receptor genes**				
14	FcεRIα	FcERI	NM_002001	F: CATGGAATCCCCTACTCTACTGTGT<br>R: CCTTAGGTTTCTGAGGGACTGCTA
15	FcεRIβ	MS4A2	NM_000139	F: GAGAAATGCAACATATCTGGTGAGAG<br>R: AGGTTGATGATCAGGATGGTAATTC
16	FcεRIγ		NM_004106	F: TCTACTGTCGACTGAAGGTAATCCA<br>R: GAGTCTCGTAAGTCTCCTGGTTCCT
17	CD23	FCER2<br>CLEC4J<br>FCE2<br>IGEBF	NM_002002	F: AGGTGTCCAGCGGCTTTGT<br>R: AGCACTTCCGTTGGAAATTGA

No	Symbol	Synonym	Acc. no.	Primer sequences
**Lineage marker genes**				
30	CD3E		NM_000733.3	F: CTGGCGGCAGGCAAAGG<br>R: TTCCGGATGGGCTCATAGTCT
31	CD14		NM_000591.3	F: CGGAAGACTTATCGACCATGGA<br>R: GACGCAGCGGAAATCTTCA
32	CD19	B4 CVID3	NM_001178098.1	F: TGACCCCACCAGGAGATTCTT<br>R: CACGTTCCCGTACTGGTTCTG
33	CD20	MS4A1<br>B1	NM_021950.3	F: CACTCTTCAGGAGGATGTCTTCAC<br>R: GACAGCCCCCAAAGTCTTAGATT
34	CD40	CDW40<br>TNFRSF5	NM_001250.4	F: TGCCAGCCAGGACAGAAAC<br>R: TGTGTCTCTCTGTTCCAGGTGTCT
35	CD80	B7-1<br>CD28LG	NM_005191.3	F: ACATCACCATCCAAGTGTCCATAC<br>R: TAACACCTGAACAGAAGTGAGAAAGAC
36	HLA-DRA		NM_019111.4	F: GCCCTGGGCCTGACTGT<br>R: CATCACCTCCATGTGCCTTACA
**Housekeeping genes**				
37	EF1A	EEF1A1	NM_001402	F: ATTACAGGGACATCTCAGGCTGAC<br>R: CATTCTTGGAGATACCAGCTTCAA
38	UBC		NM_021009	F: ATTTGGGTCGCAGTTCTTG<br>R: TGCCTTGACATTCTCGATGGT
