# Supplementary material for: The Major Birch Pollen Allergen Bet v 1 Induces Different Responses in Dendritic Cells of Birch Pollen Allergic and Healthy Individuals
Source: PLoS One. 2015 Jan 30;10(1):e0117904. doi: 10.1371/journal.pone.0117904 (PMC4311984; doi:10.1371/journal.pone.0117904)
Supplement: S3 Table — The values are mean ± standard deviation (SD) of 7 independent donors within each group. (PDF) [file pone.0117904.s003.pdf]

S3 Table. Expression pattern of lineage markers in iMoDCs of BP allergic and normal donors assayed by flow cytometry. The values are mean ± standard deviation (SD) of 7 independent donors within each group.

BP allergic group	CD3	CD14	CD20	CD16	CD56	CD11c
mean % pos. cells	1.4	0.4	8.2	75.5	0.4	87.4
± SD	0.8	0.2	1.4	8.2	0.5	3.6

Normal donor group	CD3	CD14	CD20	CD16	CD56	CD11c
mean % pos. cells	1.2	0.4	5.5	78.3	0.5	88.9
± SD	0.4	0.5	2.4	11.2	0.3	4.9
