# Supplementary material for: The Major Birch Pollen Allergen Bet v 1 Induces Different Responses in Dendritic Cells of Birch Pollen Allergic and Healthy Individuals
Source: PLoS One. 2015 Jan 30;10(1):e0117904. doi: 10.1371/journal.pone.0117904 (PMC4311984; doi:10.1371/journal.pone.0117904)
Supplement: S4 Table — cDNA was synthesized from mRNA isolated 2.5 h and 4 h after allergen stimulation, pooled and analyzed for expression of 96 genes using the Human Immune Array. Genes whose expression changed by at least 3-fold compared with the baseline levels of unstimulated cells (Bet v 1 vs. unstimulated) or MFs (Bet v 1 + MFs vs. MFs) are shown in bold. (PDF) [file pone.0117904.s004.pdf]

S4 Table. Differentially expressed genes after allergen stimulation of cells from a BP allergic (A) and a normal donor (B). cDNA was synthesized from mRNA isolated 2.5 h and 4 h after allergen stimulation, pooled and analyzed for expression of 96 genes using the Human Immune Array. Genes whose expression changed by at least 3-fold compared with the baseline levels of unstimulated cells (Bet v 1 vs. unstimulated) or MFs (Bet v 1 + MFs vs. MFs) are shown in bold.

A

	**Relative expression**	
**Gene**	**Bet v 1 vs. unstimulated**	**Bet v 1 + MFs vs. MFs**
IL3-Hs00174117_m1	**4.24**	**14.04**
IL4-Hs00174122_m1	**6.60**	**11.67**
IL5-Hs00174200_m1	1.07	**7.55**
IL13-Hs00174379_m1	**267.11**	**23.54**
CXCL10-Hs00171042_m1	**0.29**	1.03
CXCL11-Hs00171138_m1	**0.17**	2.40

B

	**Relative expression**	
**Gene**	**Bet v 1 vs. unstimulated**	**Bet v 1 + MFs vs. MFs**
IL3-Hs00174117_m1	1.13	0.80
IL4-Hs00174122_m1	1.08	2.10
IL5-Hs00174200_m1	1.13	0.80
IL13-Hs00174379_m1	1.13	**0.31**
CXCL10-Hs00171042_m1	**3.52**	1.16
CXCL11-Hs00171138_m1	**7.00**	**3.56**
