# Supplementary figures and images for: The Major Birch Pollen Allergen Bet v 1 Induces Different Responses in Dendritic Cells of Birch Pollen Allergic and Healthy Individuals
Source: PLoS One. 2015 Jan 30;10(1):e0117904. doi: 10.1371/journal.pone.0117904 (PMC4311984; doi:10.1371/journal.pone.0117904)

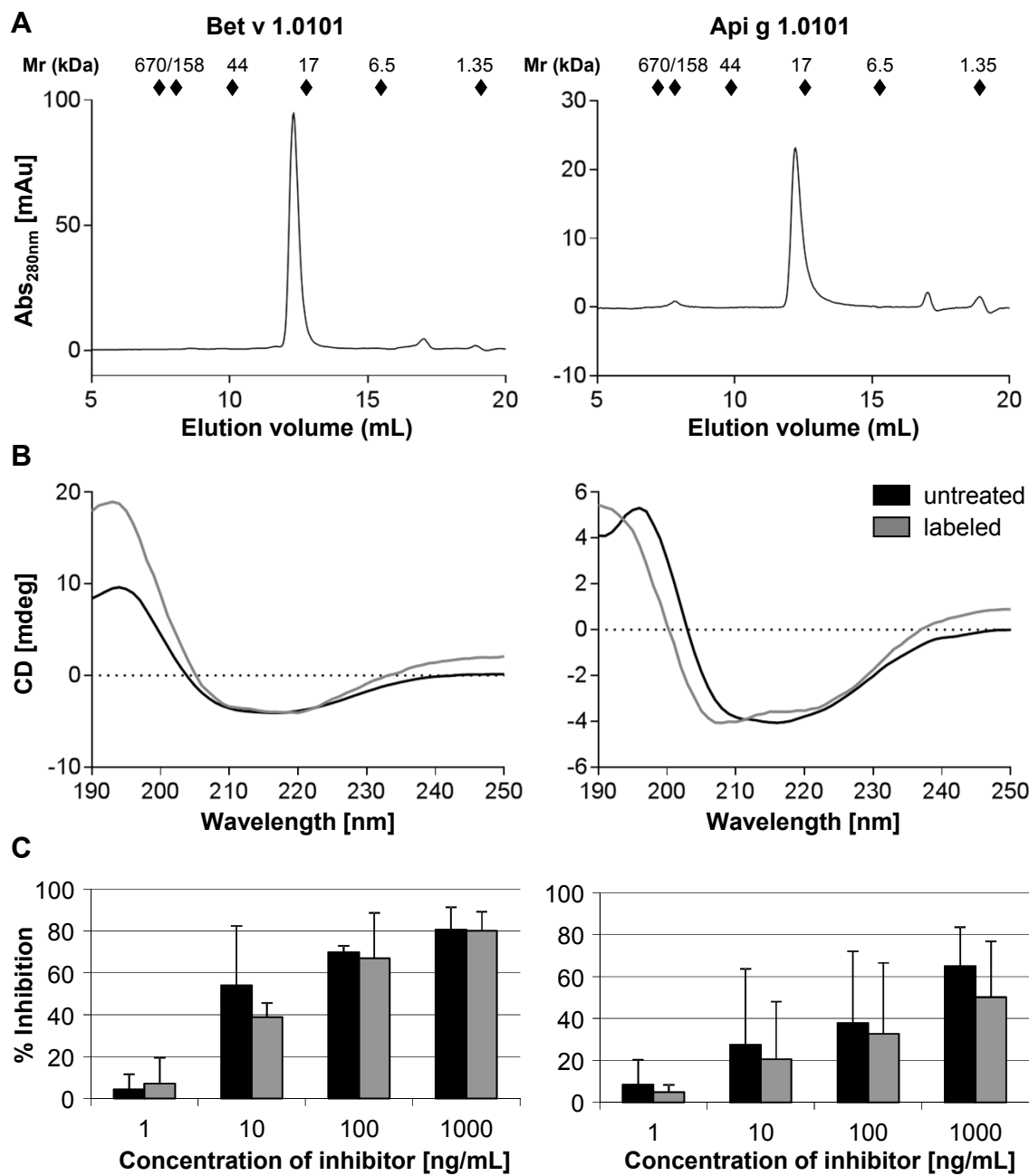

Supplement: S1 Fig — (A) Size exclusion chromatography of Bet v 1 and Api g 1. Molecular weight standards are indicated by filled diamonds. (B) CD spectra of unlabeled allergens (black lines) and Alexa-labeled allergens (grey lines) at pH 7.5. (C) Inhibition of IgE binding to Bet v 1 and Api g 1 by unlabeled (black) or labeled Bet v 1 and Api g 1 (grey). Results are represented as mean values and standard deviation of three patients’ sera. (PDF) [file pone.0117904.s005.pdf]

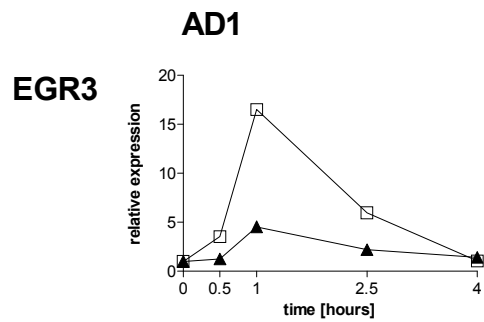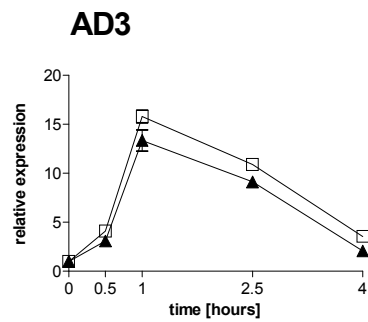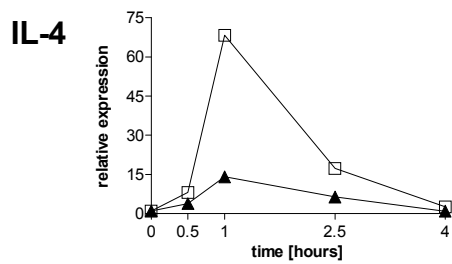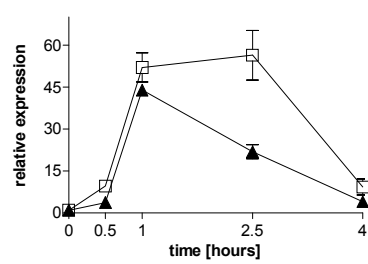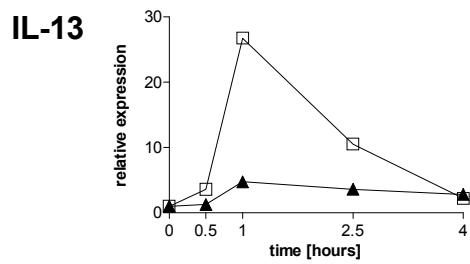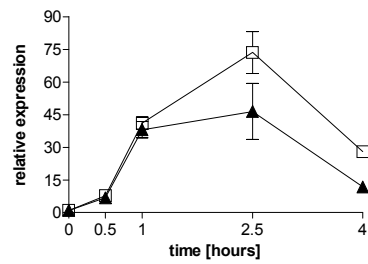

□ Bet v 1    ▲ Api g 1

Supplement: S2 Fig — Cells were sham treated or treated with Bet v 1 (squares) or Api g 1 (triangles) for indicated time periods. mRNA levels of EGR3 and the Th2 cytokines IL-4 and IL-13 were analyzed by real-time PCR. Expression levels, normalized to the average of housekeeping genes, are shown relative to unstimulated cells. Mean values and SD of duplicate experiments with cells of two donors are shown. (PDF) [file pone.0117904.s006.pdf]

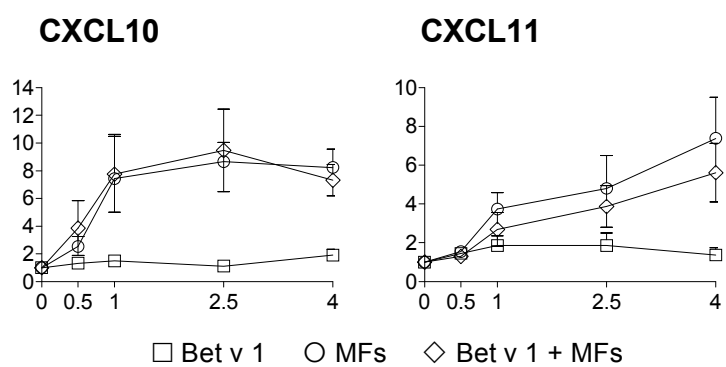

Supplement: S3 Fig — Cells were sham treated or treated with Bet v 1 (squares), a control stimulus (MFs; circles), or a combination of allergen and control stimulus (diamonds) for the indicated time periods. mRNA levels of the Th1 chemokines CXCL10 and 11 were analyzed by real-time PCR. Expression levels, normalized to the average of housekeeping genes, are shown relative to unstimulated cells. Data are presented as mean values ± SD from independent experiments using cells from four different normal donors each performed in duplicates. (PDF) [file pone.0117904.s007.pdf]

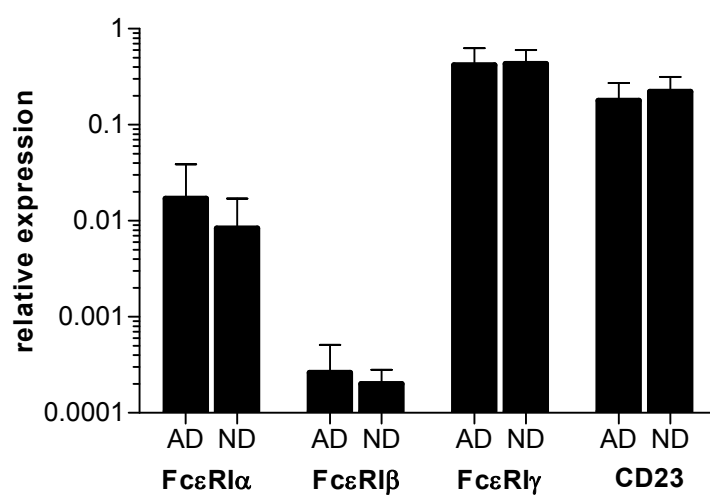

Supplement: S4 Fig — mRNA of unstimulated iMoDCs of BP allergic (AD) and healthy donors (ND) was analyzed by real-time PCR for the expression of FcεRIα, FcεRIβ, FcεRIγ, and CD23. Expression levels were normalized to the average of housekeeping genes. Values are displayed as mean values ± SD of four donors per group. (PDF) [file pone.0117904.s008.pdf]
